# A Stochastic-Variational Model for Soft Mumford-Shah Segmentation

**DOI:** 10.1155/IJBI/2006/92329

**Published:** 2006-04-12

**Authors:** Jianhong (Jackie) Shen

**Affiliations:** ^1^ School of Mathematics, Institute of Technology, University of Minnesota, Minneapolis, MN 55455, USA; ^2^ Lotus Hill Institute for Computer Vision and Information Science, E'Zhou, Wuhan 436000, China

## Abstract

In contemporary image and vision analysis, stochastic approaches demonstrate great flexibility in representing and modeling complex phenomena, while variational-PDE methods gain enormous computational advantages over Monte Carlo or other stochastic algorithms. In combination, the two can lead to much more powerful novel models and efficient algorithms. In the current work, we propose a stochastic-variational model for *soft* (or fuzzy) Mumford-Shah segmentation of mixture image patterns. Unlike the classical *hard* Mumford-Shah segmentation, the new model allows each pixel to belong to each image pattern with some probability. Soft segmentation could lead to hard segmentation, and hence is more general. The modeling procedure, mathematical analysis on the existence of optimal solutions, and computational implementation of the new model are explored in detail, and numerical examples of both synthetic and natural images are presented.


Hindawi Publishing Corporation
International Journal of Biomedical Imaging
Volume 2006, Article ID 92329, Pages 1–14
DOI 10.1155/IJBI/2006/92329

### A Stochastic-Variational Model for Soft Mumford-Shah Segmentation

**Jianhong (Jackie) Shen**[1, 2]

[1] *School of Mathematics, Institute of Technology, University of Minnesota, Minneapolis, MN 55455, USA*
[2] *Lotus Hill Institute for Computer Vision and Information Science, E'Zhou, Wuhan 436000, China*

Received 20 September 2005; Revised 13 February 2006; Accepted 17 February 2006

Recommended for Publication by Guowei Wei

In contemporary image and vision analysis, stochastic approaches demonstrate great flexibility in representing and modeling complex phenomena, while variational-PDE methods gain enormous computational advantages over Monte Carlo or other stochastic algorithms. In combination, the two can lead to much more powerful novel models and efficient algorithms. In the current work, we propose a stochastic-variational model for *soft* (or fuzzy) Mumford-Shah segmentation of mixture image patterns. Unlike the classical *hard* Mumford-Shah segmentation, the new model allows each pixel to belong to each image pattern with some probability. Soft segmentation could lead to hard segmentation, and hence is more general. The modeling procedure, mathematical analysis on the existence of optimal solutions, and computational implementation of the new model are explored in detail, and numerical examples of both synthetic and natural images are presented.

Copyright © 2006 Jianhong (Jackie) Shen. This is an open access article distributed under the Creative Commons Attribution License, which permits unrestricted use, distribution, and reproduction in any medium, provided the original work is properly cited.

### 1. INTRODUCTION: SOFT VERSUS HARD SEGMENTATION

Segmentation is the key step towards high-level vision modeling and analysis, including object characterization, detection, and classification. There have been some recent developments indicating that certain high-level visual tasks such as global scene interpretation might be able to bypass segmentation [1, 2]. Nevertheless, segmentation still remains perhaps *the* most important and inspiring task to date in low- or middle-level vision analysis and image processing.

The segmentation problem can be formulated as follows. Given an image $I \in L^2(\Omega)$ on a 2-dimensional (2D) domain $\Omega$ (assumed to be bounded, smooth, and open), one seeks out a *closed* "edge set" $\Gamma$, and all the connected components $\Omega_1, \ldots, \Omega_K$ of $\Omega \setminus \Gamma$, such that by certain suitable visual measure (e.g., textural or photometric), the image $I$ is discontinuous along $\Gamma$ while smooth or homogeneous on each segment $\Omega_i$. Each image patch $I_i = I|_{\Omega_i}$ is also called a *pattern*, and $\Omega_i$ is its *support*.

We will call this most common practice "hard" segmentation. A hard segmentation partitions the image domain $\Omega$ along a *definitive* edge set $\Gamma$, and outputs *nonoverlapping* pattern supports $\Omega_1, \ldots, \Omega_K$.

The present work introduces the notion of "soft" segmentation. Mathematically, a hard segmentation amounts to the partition of the unit using indicator functions:

$$1_\Omega(x) = \sum_{i=1}^{K} 1_{\Omega_i}(x), \quad \text{a.e. (in Lebesgue) } x = (x_1, x_2) \in \Omega. \tag{1}$$

A soft segmentation seeks out instead a softer partition of the unit:

$$1_\Omega(x) = \sum_{i=1}^{K} p_i(x), \tag{2}$$

where $p_i$'s are continuous or smoother functions. Formally, each $p_i$ could be considered as the mollified version of $1_{\Omega_i}(x)$.

In the stochastic literature of image analysis and modeling, the above notion of soft segmentation is closely connected to *mixture image models* (e.g., [3]). Suppose a given image $I$ is composed of $K$ unknown patterns:

$$\omega = 1, \omega = 2, \ldots, \omega = K, \tag{3}$$

where $\omega$ denotes the pattern label variable. At each pixel $x \in \Omega$, $\omega(x) \in \{1, \ldots, K\}$ becomes a random variable. Then the

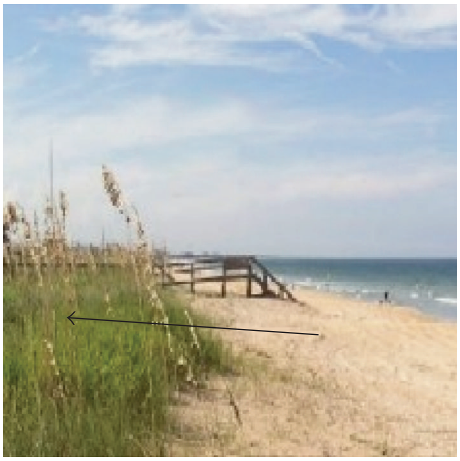

FIGURE 1: Natural images often do not have clear-cut "hard" boundaries between different patterns. Along the arrow, for example, one only observes that the sand pattern *gradually* becomes a grass pattern. Such a "soft" view is the stochastic view on the segmentation problem.

$p_i$'s in (2) carry the natural stochastic interpretation:

$$p_i(x) = \text{Prob}\,(\omega(x) = i), \quad i = 1 : K. \tag{4}$$

For this reason, each $p_i$ will be called the *ownership* of pattern $i$, following Jepson and Black [3]. (Some authors also prefer to call it the *membership* [4].) Instead of the repulsive ownership in a hard segmentation, a soft one allows each pattern to "own" a pixel with some likelihood.

Soft segmentation is more general since it can lead to natural hard segmentation under the *maximum-likelihood* (ML) principle. Given a soft segmentation $\{p_i(x) : i = 1 : K\}$, one can define for each pixel $x \in \Omega$ its unique owner $\omega_*(x)$ by

$$\omega_*(x) = \arg\max_{\omega \in 1:K} p_\omega(x), \tag{5}$$

and if the maxima are nonunique, accept the largest index from the arg max pool. The segments are then defined by

$$\Omega_i = \omega_*^{-1}(i) = \{x \in \Omega \mid \omega_*(x) = i\}, \quad i = 1 : K, \tag{6}$$

which leads to a natural hard segmentation. Formula (5) and (6) are called the *hardening formulae*.

Soft segmentation has been motivated by practical analysis of natural images. Patterns in natural scenes often do not have clear-cut boundaries. In Figure 1, for example, there does not seem to exist a "hard" boundary between the grass and sand areas. If one draws an oriented line as shown in the figure, it makes more sense to state that along the arrow, the pattern transits from being "more" sand-like to being "more" grass-like. Such consideration favors the following stochastic view that along the arrow, the ownership

$$\begin{aligned} &\text{Prob}\,(\omega(x) = \text{grass}) \text{ increases,} \\ &\text{while Prob}\,(\omega(x) = \text{sand}) \text{ decreases.} \end{aligned} \tag{7}$$

In the present work, we propose a new stochastic-variational soft segmentation model for the following celebrated Mumford-Shah model [5, 6]:

$$\min_{\Gamma,u} E[u,\Gamma \mid I] = \min_{\Gamma,u} \mathcal{H}^1(\Gamma) + \alpha \int_{\Omega\setminus\Gamma} |\nabla u|^2 + \lambda \int_\Omega (u - I)^2, \tag{8}$$

where $\mathcal{H}^1$ stands for the 1D Hausdorff measure [7], which is simply the length when $\Gamma$ is regular enough. *For notational conciseness, the default area-element symbol $dx = dx_1 dx_2$ will be omitted in most integral formulae.*

As stated in the abstract, the stochastic softness induces more flexibility and universality in modeling, while the variational-PDE approach facilitates rigorous mathematical analysis as well as more efficient computational implementations compared with purely stochastic approaches including, for example, the Monte Carlo method or Gibbs' sampling [8–11].

The paper has been organized as follows. Section 2 builds up the soft Mumford-Shah (SMS) model under the Bayesian rationale and the MAP estimator [12, 13], which are the formal stochastic foundations of the present model. In Section 3, the prior energy on the ownerships $p_i$'s is developed based on the celebrated work of Modica and Mortola [14] on phase-field modeling and Γ-convergence approximation in material sciences and phase transitions. In Section 4, we analyze the main mathematical properties of the proposed SMS model, including the admissible space, hidden symmetry and symmetry breaking via weak supervision, and the existence theorems. In Section 5, we then derive the system of Euler-Lagrange equations of the SMS model for which the role of the probability simplex constraint is discussed in detail. Section 5 also introduces the alternating-minimization algorithm to compute the Euler-Lagrange equations. Finally, the numerical performance of the SMS model is demonstrated in Section 6 via both synthetic and natural test images that are sufficiently representative and generic.

Throughout the manuscript, the notation $F[X, Y \mid Z]$ in the deterministic setting always denotes a quantity (often a functional, or an energy) $F$ that depends on $X$, $Y$, and $Z$ but with $Z$ given or fixed. Similarly, $F[X \mid Y, Z]$ still denotes $F[X, Y \mid Z]$ modulo some additive quantity $g[Y, Z]$ that is often unimportant as far as the optimization on $X$ (given $Y$ and $Z$) is concerned. These notations therefore have been inspired by *conditional probabilities* in the stochastic setting (formally under the Gibbs' correspondence: $F[X \mid Y] = -\log p(X \mid Y)$).

### 2. BAYESIAN RATIONALE TO THE NEW MODEL AND GAUSSIAN MIXTURE

### 2.1. *Bayesian rationale*

Segmentation can be done in some feature spaces such as gradient-like highpass features or Gabor features (e.g., [11, 15, 16]). The Mumford-Shah model easily extends to such general features (e.g., [15]), even though it was originally formulated only for intensity fields. For maximal clarity in exposing the core ideas of the current work, we will also focus only on the latter, while leaving as canonical exercises to adapt the new model for any given feature distribution.

Let $K$ be the total number of intended patterns. As in [10, 11], $K$ could also be treated as an unknown to be optimally estimated, which however does not add much to the most significant contribution (i.e., the modeling and computation of the "softening" procedure) of the present work.

Given an image input $I = I(x)$ on a bounded, regular, and open domain $\Omega$, the primary goal of soft segmentation is to compute the ownerships

$$p_1(x), p_2(x), \dots, p_K(x). \tag{9}$$

Define $\mathbf{P}(x) = (p_1(x), p_2(x), \dots, p_K(x))$, and

$$\Delta_{K-1} = \text{convex hull of } \vec{e}_1, \dots, \vec{e}_K, \tag{10}$$

where the $(\vec{e}_i \mid i = 1 : K)$ denotes the canonical Cartesian basis of $\mathbb{R}^K$. $\Delta_{K-1}$ is often called the *canonical* $(K-1)$-simplex, or the *probability* simplex in $\mathbb{R}^K$. Then

$$\mathbf{P} : \Omega \longrightarrow \Delta_{K-1}, \qquad x \longrightarrow \mathbf{P}(x), \tag{11}$$

meaning that the total ownerships always add up to 100% at any pixel $x \in \Omega$.

Associated with each pattern label, $\omega = i$ is a smooth function $u_i(x) \in H^1(\Omega)$, similar to the original Mumford-Shah model. Here the Sobolev space $H^1(\Omega)$ is defined by [17]

$$H^1(\Omega) = \{u \in L^2(\Omega) \mid \nabla u \in L^2(\Omega, \mathbb{R}^2)\}. \tag{12}$$

Define $\mathbf{U}(x) = (u_1(x), u_2(x), \dots, u_K(x))$. Then the goal of soft segmentation is to estimate the optimal vectorial pair of ownerships and patterns given an image $I$:

$$(\mathbf{P}_*, \mathbf{U}_*) = \underset{(\mathbf{P},\mathbf{U})}{\arg\max} \operatorname{Prob}(\mathbf{P}, \mathbf{U} \mid I). \tag{13}$$

By the Bayesian formula [12, 13], the posterior given $I$ is expressible via

$$\frac{\operatorname{Prob}(\mathbf{P}, \mathbf{U} \mid I) = \operatorname{Prob}(I \mid \mathbf{P}, \mathbf{U}) \operatorname{Prob}(\mathbf{P}) \operatorname{Prob}(\mathbf{U})}{\operatorname{Prob}(I)}, \tag{14}$$

assuming that the mixture patterns $\mathbf{U}$ and the mixture rules $\mathbf{P}$ are *independent* (as two vectorial random fields). We will call the first term a "mixture generation" model, since it reveals how the image data should look like given the information of the patterns and their ownerships.

By taking logarithmic likelihood $E[\cdot] = -\log \operatorname{Prob}(\cdot)$, or the formally Gibbs' energy in statistical mechanics [18, 19], one attains the soft segmentation model in its "energy" form:

$$\begin{aligned} &\underset{(\mathbf{P},\mathbf{U})}{\arg\min}\, E[\mathbf{P}, \mathbf{U} \mid I] \\ &\quad = \underset{(\mathbf{P},\mathbf{U})}{\arg\min}\, E[I \mid \mathbf{P}, \mathbf{U}] + E[\mathbf{P}] + E[\mathbf{U}]. \end{aligned} \tag{15}$$

Assuming that all the pattern channels are independent of each other, one has

$$E[\mathbf{U}] = E[u_1, \dots, u_K] = \sum_{i=1}^{K} E[u_i \mid i]. \tag{16}$$

That is, we assume that $\mathbf{U}$ as a random vector field has independent scalar components. It has been motivated by the facts that 2D images are the optical projections of 3D scenes and that different objects in 3D are independently positioned in different ranges or depths.

For Sobolev-regular patterns, that is, functions whose gradients are square integrable, one may impose the homogeneous Sobolev energies:

$$E[u_i \mid i] = E[u_i] = \alpha \int_\Omega |\nabla u_i|^2, \quad i = 1 : K, \tag{17}$$

for some scalar weight $\alpha$ that models the visual sensitivity to intensity roughness. Unlike the original Mumford-Shah model, the energy for each channel has been defined on the entire image domain $\Omega$ instead of on each "hard-cut" patch $\Omega_i$. Thus the energy form (17) must carry out *extrapolation* for practical applications. Long-range extrapolations are, however, often unimportant after being weighed down by their negligible ownerships $p_i$'s.

### 2.2. Gaussian mixture with smooth mean fields

In this section we discuss the mixture generation model $\operatorname{Prob}(I \mid \mathbf{P}, \mathbf{U})$ or $E[I \mid \mathbf{P}, \mathbf{U}]$.

Assume that the patterns are all Gaussian with mean fields $u_1, u_2, \dots, u_K$. For simplicity, also assume that they share the same variance $\sigma^2$ (which readily generalizes to the more general case with variations). Then at any given pixel $x \in \Omega$,

$$(I \mid \omega(x) = i) \sim N(u_i(x), \sigma^2), \quad i = 1 : K. \tag{18}$$

Define the Gaussian probability density function (pdf)

$$g(I \mid m, \sigma) = \frac{1}{\sqrt{2\pi}\sigma} \exp\left(-\frac{(I-m)^2}{2\sigma^2}\right). \tag{19}$$

The pdf of the mixture image $I$ at any pixel $x$ is given by

$$\begin{aligned} &\operatorname{Prob}(I(x) \mid \mathbf{P}(x), \mathbf{U}(x)) \\ &\quad = \sum_{i=1}^{K} \operatorname{Prob}(I \mid \omega(x) = i) \operatorname{Prob}(\omega(x) = i) \\ &\quad = \sum_{i=1}^{K} g(I \mid u_i(x), \sigma)\, p_i(x). \end{aligned} \tag{20}$$

Thus *ideally* the "energy" for the mixture generation model should be given by

$$\begin{aligned} E[I \mid \mathbf{P}, \mathbf{U}] &= E_\mu[I \mid \mathbf{P}, \mathbf{U}] \\ &= -\mu \int_\Omega \log\left(\sum_{i=1}^{K} g(I \mid u_i(x), \sigma)\, p_i(x)\right), \\ &\qquad \text{for some } \mu > 0, \end{aligned} \tag{21}$$

provided that given two fields $\mathbf{P}$ and $\mathbf{U}$ on $\Omega$, for any two disjoint and finite sets of pixels $X$ and $Y$,

$$(I(X) \mid \mathbf{P}, \mathbf{U}) \text{ is independent of } (I(Y) \mid \mathbf{P}, \mathbf{U}). \tag{22}$$

Here $I(X) = \{I(x) \mid x \in X\}$. (We also must emphasize that the above derivation should be considered as motivational rather than rigorous, due to the continuum setting.)

In the current work, we will adopt a reduced form of the complex formula (21), which is simpler and easier to manage both in theory and for computation. Assume that each soft ownership $p_i(x)$ is closer to a hard one $p_i(x) \simeq 1_{\Omega_i}(x)$ for $i = 1 : K$. Then

$$\begin{aligned} -\log\left(\sum_{i=1}^{K} g(I \mid u_i(x), \sigma)\, p_i(x)\right) &\simeq -\log\left(\sum_{i=1}^{K} g(I \mid u_i(x), \sigma)\, 1_{\Omega_i}(x)\right) \\ &= -\sum_{i=1}^{K} \log g(I \mid u_i(x), \sigma)\, 1_{\Omega_i}(x) \quad \text{(a.e.)} \\ &\simeq -\sum_{i=1}^{K} \log g(I \mid u_i(x), \sigma)\, p_i(x) \\ &= \frac{1}{2\sigma^2} \sum_{i=1}^{K} (I - u_i(x))^2 p_i(x) + \text{const}, \end{aligned} \tag{23}$$

where the additive constant only depends on $\sigma$ and $K$. This suggests the following convenient energy form for the mixture generation model:

$$E[I \mid \mathbf{P}, \mathbf{U}] = \lambda \int_{\Omega} \left(\sum_{i=1}^{K} (I - u_i(x))^2 p_i(x)\right), \tag{24}$$

which amounts to a weighted least-square energy [20]. The weight $\lambda$ reflects visual sensitivity to synthesis errors.

In combination of (15), (17), and (24), the new soft segmentation model takes the form of minimizing

$$\begin{aligned} E[\mathbf{P}, \mathbf{U} \mid I] = \lambda \sum_{i=1}^{K} \int_{\Omega} (I - u_i(x))^2 p_i(x) \\ + \alpha \sum_{i=1}^{K} \int_{\Omega} |\nabla u_i|^2 + E[\mathbf{P}]. \end{aligned} \tag{25}$$

Notice that here the ownership distribution $\mathbf{P}$ "softens" the "hard" segmentation boundary $\Gamma$ in the original Mumford-Shah model (8). To complete the modeling process, it suffices to properly define the prior or regularity energy $E[\mathbf{P}]$, which is the main task of the next section.

### 3. MODICA-MORTOLA'S PHASE-FIELD MODEL FOR OWNERSHIP ENERGY

To generalize but not to deviate too far from classical hard segmentation, it is natural to impose the following two constraints:

(a) each pattern ownership $p_i(x)$ has almost only two phases: on (corresponding to $p_i = 1$) and off (to $p_i = 0$), and the transition band in between is narrow;

(b) the soft boundaries, or equivalently the transition bands, are *regular*, instead of being zigzag.

In combination, one imposes the following Modica-Mortola type of energy with a double-well potential [14]: $p_i \in H^1(\Omega)$,

$$E_\varepsilon[p_i] = \int_{\Omega} \left(9\varepsilon |\nabla p_i|^2 + \frac{(p_i(1 - p_i))^2}{\varepsilon}\right), \quad i = 1 : K. \tag{26}$$

Here $\varepsilon \ll 1$ controls the transition bandwidth. Since $\varepsilon \ll 1$, the second term necessarily demands $p_i \simeq 0$ or 1 to lower the energy, which well resonates with the expectation in (a). The first term, weighted by the small parameter $\varepsilon$, amounts to a regularity condition on each $p_i$, which meets the requirement in (b).

Energies in the form of (26) are very common in material sciences, including the theories of liquid crystals and phase transitions [21, 22]. Mathematically, they have been well studied in the framework of $\Gamma$-convergence [23], which we now give a brief introduction in the present context. We also refer the reader to the works of Ambrosio and Tortorelli [24, 25] on the $\Gamma$-convergence approximation to the classical Mumford-Shah segmentation model.

Recall that for any $q(x) \in L^1(\Omega)$, its *total variation* as a Radon measure is defined by [7, 26, 27]

$$\text{TV}[q] = \int_{\Omega} |Dq| = \sup_{\mathbf{g} \in C_0^1(\Omega, B^2)} \langle q, \nabla \cdot \mathbf{g} \rangle, \tag{27}$$

where $B^2$ stands for the unit disk centered at the origin in $\mathbb{R}^2$. (The TV measure was first introduced into image processing by Rudin et al. [28].) Define that for any $q \in L^1(\Omega)$,

$$E_0[q] = \begin{cases} \text{TV}[q] & \text{if } q = 0 \text{ or } 1, \text{a.e. on } \Omega, \\ \infty & \text{otherwise.} \end{cases} \tag{28}$$

As a result, a finite energy $E_0[q]$ necessarily implies that $q$ has two phases only, and $E_0[q] = \text{TV}[q] = \text{Per}(q^{-1}(1))$ is the perimeter of the support region $V = q^{-1}(1)$.

Further define

$$L^1_{[0,1]}(\Omega) = \{q \in L^1(\Omega) \mid q(x) \in [0, 1],\ \forall x \in \Omega\} \tag{29}$$

to be a subspace of $L^1(\Omega)$ (as a metric space). Then Modica and Mortola's well-known results in [14] readily lead to the following theorem.

**Theorem 1** ($\Gamma$-convergence approximation of a two-phase TV). *For any $q \in L^1_{[0,1]}(\Omega) \setminus H^1(\Omega)$, extend the definition of $E_\varepsilon[\cdot]$ in (26) by defining $E_\varepsilon[q] = +\infty$. Then*

$$E_\varepsilon \longrightarrow E_0 \quad \textit{in the sense of } \Gamma\textit{-convergence in the metric space } L^1_{[0,1]}(\Omega). \tag{30}$$

*That is,*

(i) *for any $q_\varepsilon \to q$ in $L^1_{[0,1]}(\Omega)$ as $\varepsilon \to 0$,*

$$\liminf_{\varepsilon \to 0} E_\varepsilon[q_\varepsilon] \geq E_0[q]; \tag{31}$$

(ii) *for any* $q \in L^1_{[0,1]}(\Omega)$, *there exists some sequence* $(q^*_\varepsilon \mid \varepsilon)$, *such that* $q^*_\varepsilon \to q$ *as* $\varepsilon \to 0$, *and*

$$\lim_{\varepsilon\to 0} E_\varepsilon[q^*_\varepsilon] = E_0[q]. \tag{32}$$

We refer the reader to Modica and Mortola [14] for a proof (with some necessary modification). Here we only point out that the "tight" sequence $(q^*_\varepsilon \mid \varepsilon)$ in (ii) can be constructed using a smooth sigmoid transition across the hard boundary of a given two-phase function $q$. Recall as in the theory of neural networks [29] that a *sigmoid* transition between 0 and 1 is achieved by

$$\sigma(t) = \frac{1}{1+e^{-t}}, \quad -\infty < t < \infty. \tag{33}$$

The scaling parameter $\varepsilon$ participates in the transition by the form of $\sigma(t/(3\varepsilon))$. In particular, $\varepsilon$ indeed corresponds to the width of the transition band when $t$ is a distance function.

This theorem reveals the close connection of the particular choice of $E_\varepsilon[p_i]$ in (26) with the original Mumford-Shah model.

**Proposition 1.** *Suppose that* $p_\varepsilon$*'s "optimally" (i.e., by the above sigmoidal transition) converge to a given 2-phase pattern* $1_V(x)$ *with a regular hard boundary* $\Gamma = \partial V$. *Then,*

$$E_\varepsilon[p_\varepsilon] \longrightarrow \mathit{length}(\Gamma) = \int_\Omega |D1_V(x)|. \tag{34}$$

Similar results have appeared in the earlier influential works of Ambrosio and Tortorelli [24, 25] on the Γ-convergence approximation to the Mumford-Shah model. The technique has also been extensively applied in image computation and modeling [30–35] to overcome the difficulty in representing and computing the free boundary Γ.

To summarize this section, we propose the following energy model for the ownership distribution $\mathbf{P}(x) = (p_1(x), p_2(x), \dots, p_K(x))$:

$$E_\varepsilon[\mathbf{P}] = \sum_{i=1}^K E_\varepsilon[p_i] = \sum_{i=1}^K \int_\Omega \left(9\varepsilon|\nabla p_i|^2 + \frac{(p_i(1-p_i))^2}{\varepsilon}\right). \tag{35}$$

One, however, must realize that different ownerships are *not* decoupled by this energy though it has appeared so. The energy $E_\varepsilon[\mathbf{P}]$ must be coupled with the constraint of the probability simplex:

$$\mathbf{P}:\Omega \longrightarrow \Delta_{K-1}, \quad \text{or} \quad \sum_{i=1}^K p_i(x) \equiv 1, \quad p_i \ge 0, \ \forall x \in \Omega. \tag{36}$$

In particular, for small $\varepsilon$, although (35) implies that each ownership $p_i$ tends to polarize to 0 or 1 independently, they have to cooperate with each other under the above simplex constraint to optimally share the ownerships.

### 4. SOFT MUMFORD-SHAH SEGMENTATION

### 4.1. The model and admission space

Combining the preceding two sections, we have developed the complete formula for soft Mumford-Shah segmentation with $K$ patterns, that is, to minimize

$$E[\mathbf{P},\mathbf{U} \mid I] = \lambda \sum_{i=1}^K \int_\Omega (u_i - I)^2 p_i + \alpha \sum_{i=1}^K \int_\Omega |\nabla u_i|^2 + \sum_{i=1}^K \int_\Omega \left(9\varepsilon|\nabla p_i|^2 + \frac{(p_i(1-p_i))^2}{\varepsilon}\right), \tag{37}$$

with the constraint that

$$\mathbf{P}:\Omega \longrightarrow \Delta_{K-1}, \quad \text{the probability } (K-1)\text{-simplex}, \tag{38}$$

that is, $p_i \ge 0$, $i = 1 : K$, and $\sum_{i=1}^K p_i = 1$. As discussed previously, it is this simplex constraint that induces coupling among different channels into the seemingly decoupled model (37).

Besides the simplex constraint, the last term in the energy (37) requires $p_i \in H^1(\Omega)$ for $i = 1 : K$. Similarly, the second term requires each pattern $u_i \in H^1(\Omega)$. Then with the assumption that

$$\text{"the given image } I \in L^2(\Omega)\text{,"} \tag{39}$$

$E[\mathbf{P},\mathbf{U} \mid I]$ is well defined and finite for any admissible patterns $\mathbf{U}$ and pattern ownership distribution $\mathbf{P}$:

$$\text{adm}_K = \{(\mathbf{P},\mathbf{U}) \mid p_i,\ u_i \in H^1(\Omega),\ i=1:K;\ \mathbf{P}:\Omega \longrightarrow \Delta_{K-1}\}. \tag{40}$$

### 4.2. Breaking the hidden symmetry via weak supervision

Let $S_K$ denote the permutation group of $\{1,\dots,K\}$. Each permutation $\sigma \in S_K$ is a 1-to-1 map:

$$\sigma : \{1,\dots,K\} \longrightarrow \{1,\dots,K\}, \tag{41}$$

so that $(\sigma(1),\dots,\sigma(K))$ is a rearrangement of $\{1,\dots,K\}$. For any $K$-tuple $F = (f_1,\dots,f_K)$, one defines

$$F_\sigma = (f_{\sigma(1)}, f_{\sigma(2)}, \dots, f_{\sigma(K)}). \tag{42}$$

**Theorem 2** (hidden symmetry of SMS). *For any* $\sigma \in S_K$,

$$E[\mathbf{P}_\sigma, \mathbf{U}_\sigma \mid I] = E[\mathbf{P},\mathbf{U} \mid I]. \tag{43}$$

*In particular, suppose that*

$$(\mathbf{P}^*, \mathbf{U}^*) = \mathop{\arg\min}_{(\mathbf{P},\mathbf{U})\in \text{adm}_K} E[\mathbf{P},\mathbf{U} \mid I] \tag{44}$$

*is an optimal pair. Then for any* $\sigma \in S_K$, $(\mathbf{P}^*_\sigma,\ \mathbf{U}^*_\sigma)$ *is a minimizer as well.*

The proof is straightforward and thus omitted. Such symmetry not only worsens the nonuniqueness of the minima to the nonconvex energy functional in (37), but also potentially jitters intermediate solutions in iterative computational schemes (i.e., hysterical transitions in the admissible space).

To break the permutation symmetry, we turn to a weak supervision scheme in which a user specifies $K$ distinct domain patches:

$$Q_1, Q_2, \ldots, Q_K, \tag{45}$$

and imposes the symmetry-breaking conditions:

$$p_i|_{Q_j} = \delta_{ij}, \quad i, j = 1 : K, \tag{46}$$

where $\delta_{ij}$ denotes Kronecker's delta. That is, a user requires each given patch $Q_i$ to be a "pure" pattern exclusively labelled by $i$. Computationally, this weak supervision process can be automated based on multiscale patch statistics as in the contemporary works on scene recognition [1, 2], or more generally, the learning theory [36, 37].

### *4.3. Existence theorems for nonsupervision and supervision*

In this section, we briefly state the existence theorems for the soft Mumford-Shah segmentation model (37) without or with the supervision (46). The detailed proof has been moved to the appendix, under the suggestion of one of our referees. Skipping this section will cause no serious problem in comprehending or implementing the models.

**Theorem 3** (existence theorem for unsupervised SMS). *Suppose that $I \in L^2(\Omega)$. Then for any positive modeling parameters $(\lambda, \alpha, \varepsilon)$, a minimizer to the unsupervised soft Mumford-Shah model* (37) *must exist.*

Mathematically, the existence issue has special appeal to model developers, especially for models that are highly nonconvex. Nonconvex variational models normally fail to guarantee the uniqueness of optimal solutions, and existence is hence often the best one can attempt to establish theoretically [38]. Notice that most interesting variational models in contemporary image and visual analysis are nonconvex, which include, for example, the original Mumford-Shah model [6], various image restoration models (e.g., deblurring and dejittering) [7, 39], as well as most optical-flow models [38].

The theoretical proof in the appendix, however, does reveal an important behavior of the model (37) which carries practical implications. If certain channel $i$ becomes dumb in the limiting process of the proof (i.e., the limit $p_i^* \equiv 0$ for a minimizing sequence), it has often been introduced unnecessarily in the first place, and the associated optimal pattern $u_i^*$ could be any featureless constant image.

The related issue of determining an *optimal* class number $K$ (i.e., without containing dumb channels nor missing visually important channels) is also intrinsically driven by the complexity theory of natural images, in particular, the multiscale complexity [40]. Theoretically, $K$ could be any integer, ranging from zero to the infinity, as one zooms into the details of a continuum image from the atomic scale to the ordinary observational scales of the naked eyes. Thus ideally, $K$ itself could be introduced as a random variable taking $0, 1, 2, \ldots$, and becomes part of the model itself. This idea has already been explored in purely stochastic settings, for example, see Tu and Zhu [10].

For the supervised scenario motivated earlier, the following existence theorem still holds.

**Theorem 4.** *Suppose that $I \in L^2(\Omega)$. Then an optimal pattern-ownership pair must exist to the soft Mumford-Shah segmentation model* (37) *with supervision* (46), *assuming that each patch $Q_i$ has a positive Lebesgue measure $|Q_i| > 0$.*

The proof is almost identical to the unsupervised case in the appendix, and simplifies substantially by noticing that no channel could become dumb due to supervision. Furthermore, the functions $\rho_i$'s in the previous proof can be directly set to be

$$\rho_i = \frac{1}{|Q_i|} 1_{Q_i}(x), \quad i = 1 : K, \tag{47}$$

without the necessity of turning to Lemma 2.

### *4.4. Mixture of homogeneous Gaussians*

When each pattern $i$ is a homogeneous Gaussian $N(m_i, \sigma)$ with a distinct mean value $m_i$, one has

$$u_i(x) \equiv m_i, \quad x \in \Omega, \ i = 1 : K. \tag{48}$$

Define $\mathbf{m} = (m_1, \ldots, m_K)$. As a result, the soft Mumford-Shah model (37) simplifies to

$$\min_{(\mathbf{P},\mathbf{m})} E[\mathbf{P}, \mathbf{m} \mid I] = \min_{(\mathbf{P},\mathbf{m})} \lambda \sum_{i=1}^{K} \int_\Omega (I - m_i)^2 p_i + \sum_{i=1}^{K} \int_\Omega \left( 9\varepsilon |\nabla p_i|^2 + \frac{(p_i(1-p_i))^2}{\varepsilon} \right). \tag{49}$$

**Theorem 5.** *Suppose that $I \in L^2(\Omega)$. Then a minimizer pair $(\mathbf{P}^*, \mathbf{m}^*)$ to $E[\mathbf{P}, \mathbf{m} \mid I]$ exists for both the unsupervised and supervised cases.*

The proof can be derived readily from the previous general cases and is hence left out. When $K = 2$, a similar model was proposed earlier by Shen [33] under the symmetrization transform:

$$p_1(x) = \frac{1 - z(x)}{2}, \quad p_2(x) = \frac{1 + z(x)}{2}, \quad z \in [-1, 1]. \tag{50}$$

The model (49) could be considered as the soft version of Chan-Vese model [41] from the point of view of region-based active contours. Chan and Vese have demonstrated that such a piecewise constant Mumford-Shah model (or the

CV model as popularly referred to in the present literature) is already powerful enough for a number of applications including medical imaging.

### 5. EULER-LAGRANGE EQUATIONS AND COMPUTATION ON $(K-1)$-SIMPLEX

### 5.1. Euler-Lagrange equations on $(K-1)$-simplex

To minimize the energy for the soft Mumford-Shah segmentation

$$E[\mathbf{P},\mathbf{U}\mid I]=\lambda\sum_{i=1}^{K}\int_{\Omega}(u_i-I)^2p_i+\alpha\sum_{i=1}^{K}\int_{\Omega}|\nabla u_i|^2 + \sum_{i=1}^{K}\int_{\Omega}\left(9\varepsilon|\nabla p_i|^2+\frac{(p_i(1-p_i))^2}{\varepsilon}\right), \tag{51}$$

one resorts to its gradient-descent flow or Euler-Lagrange equations. In this section, we discuss these equations and their practical computational schemes.

The first-order partial variation on $\mathbf{U}$ given $\mathbf{P}$ leads to, for $i=1:K$,

$$\alpha\Delta u_i+\lambda(I-u_i)p_i=0,\quad \text{on }\Omega;\qquad \frac{\partial u_i}{\partial\mathbf{n}}=0,\quad \text{along }\partial\Omega, \tag{52}$$

where $\mathbf{n}$ stands for the outer normal vector field along $\partial\Omega$. Thus the Euler-Lagrange equations on the patterns are all in the form of linear Poisson equations with variable coefficient fields:

$$-\alpha\Delta u_i+(\lambda p_i)u_i=f_i,\quad i=1:K, \tag{53}$$

with Neumann adiabatic boundary conditions, where the source terms are $f_i(x)=\lambda p_i(x)I(x)$.

The first-order variation on the ownerships $\mathbf{P}$ is carried out on the probability $(K-1)$-simplex $\Delta_{K-1}$, which is a compact manifold (with border) of codimension 1 embedded in $\mathbb{R}^K$. Chan and Shen [42] developed a general framework for modeling and computing image features that "live" on general manifolds, and especially those that are embedded in $\mathbb{R}^K$. We will follow the approach there.

Without the simplex constraint on the ownerships, for any given $\mathbf{U}$, the first-order variation of the soft energy $E$ under $\mathbf{P}\to\mathbf{P}+\delta\mathbf{P}$ is given by

$$\delta E=\int_{\Omega}\sum_{i=1}^{K}V_i\delta p_i\,dx+\int_{\partial\Omega}\sum_{i=1}^{K}v_i\delta p_i\,d\mathcal{H}^1, \tag{54}$$

where $\mathcal{H}^1$ is the 1D Hausdorff measure along $\partial\Omega$, and

$$V_i=\lambda(u_i-I)^2-18\varepsilon\Delta p_i+2\varepsilon^{-1}p_i(1-p_i)(1-2p_i), \tag{55}$$

$$v_i=18\varepsilon\frac{\partial p_i}{\partial\mathbf{n}},\quad \text{along }\partial\Omega. \tag{56}$$

Define $\mathbf{V}=(V_1,\dots,V_K)$ and $\mathbf{v}=(v_1,\dots,v_K)$. Then

$$\delta E=\int_{\Omega}\mathbf{V}\cdot\delta\mathbf{P}\,dx+\int_{\partial\Omega}\mathbf{v}\cdot\delta\mathbf{P}\,d\mathcal{H}^1, \tag{57}$$

which holds for any *free* variation of $\mathbf{P}$ in $\mathbb{R}^K$, or one writes in the free-gradient form

$$\frac{\partial E}{\partial_f\mathbf{P}}=\mathbf{V}|_{\Omega}+\mathbf{v}|_{\partial\Omega}. \tag{58}$$

In reality, $\mathbf{P}\in\Delta_{K-1}$. Let $T_{\mathbf{P}}\Delta_{K-1}$ denote the tangent space of $\Delta_{K-1}$ at any single point $\mathbf{P}\in\Delta_K$, and

$$\pi: T_{\mathbf{P}}\mathbb{R}^K\longrightarrow T_{\mathbf{P}}\Delta_{K-1} \tag{59}$$

the orthogonal projection onto the tangent space in $\mathbb{R}^K$. Since the normal direction of the tangent plane is given by $\mathbf{1}_K/\sqrt{K}=(1,\dots,1)/\sqrt{K}$, the projection operator is explicitly given by, for any $\mathbf{w}\in T_{\mathbf{P}}\mathbb{R}^K$,

$$\pi(\mathbf{w})=\mathbf{w}-\frac{\mathbf{1}_K\langle\mathbf{w},\mathbf{1}_K\rangle}{K}=\mathbf{w}-\langle\mathbf{w}\rangle\mathbf{1}_K,\quad \text{with }\langle\mathbf{w}\rangle=\frac{1}{K}\sum_{i=1}^{K}w_i. \tag{60}$$

The constrained gradient of $E$ on $\Delta_{K-1}$ is therefore given by

$$\frac{\partial E}{\partial\mathbf{P}}=\pi\left(\frac{\partial E}{\partial_f\mathbf{P}}\right)=(\mathbf{V}-\langle\mathbf{V}\rangle\mathbf{1}_K)|_{\Omega}+(\mathbf{v}-\langle\mathbf{v}\rangle\mathbf{1}_K)|_{\partial\Omega}. \tag{61}$$

In particular, the system of Euler-Lagrange equations on $\mathbf{P}$ given $\mathbf{U}$ is given by

$$\begin{aligned}V_i(x)-\langle\mathbf{V}\rangle(x)&=0,\quad x\in\Omega,\\ v_i(z)-\langle\mathbf{v}\rangle(z)&=0,\quad z\in\partial\Omega,\end{aligned} \tag{62}$$

for $i=1:K$. The coupling among different channels is evident from these two formulae.

**Lemma 1.** *Suppose that $\mathbf{P}:\Omega\to\Delta_{K-1}$. Then for any $z\in\partial\Omega$, $\langle\mathbf{v}\rangle(z)=0$, where the boundary "flux" $\mathbf{v}$ is defined in* (56).

*Proof.* This is obtained by direct computation: at any $z\in\partial\Omega$,

$$\begin{aligned}\langle\mathbf{v}\rangle&=\frac{1}{K}\sum_{i=1}^{K}v_i=\frac{18\varepsilon}{K}\sum_{i=1}^{K}\frac{\partial p_i}{\partial\mathbf{n}}\\ &=\frac{18\varepsilon}{K}\frac{\partial}{\partial\mathbf{n}}\left(\sum_{i=1}^{K}p_i\right)=\frac{18\varepsilon}{K}\frac{\partial 1}{\partial\mathbf{n}}=0.\end{aligned} \tag{63}$$

□

As a result, the boundary conditions in (62) simplify to the ordinary Neumann conditions $\partial p_i/\partial\mathbf{n}=0, i=1:K$. Combining all the above derivations, we have established the following theorem.

**Theorem 6** (Euler-Lagrange equations). *The system of Euler-Lagrange equations of $E[\mathbf{P},\mathbf{U}\mid I]$ is given by*

$$\begin{aligned}-\alpha\Delta u_i+(\lambda p_i)u_i&=(\lambda p_i)I,\\ -18\varepsilon\Delta p_i+2\varepsilon^{-1}p_i(1-p_i)(1-2p_i)&=\langle\mathbf{V}\rangle-\lambda(u_i-I)^2,\quad i=1:K,\end{aligned} \tag{64}$$

*on $\Omega$, all with Neumann boundary conditions along $\partial\Omega$. Here* $\mathbf{V} = \mathbf{V}(\mathbf{P}, \mathbf{U})$ *is defined as in* (55)*. Furthermore, under supervision* (46)*, the ownerships must satisfy the interpolation conditions:*

$$p_i|_{Q_j} = \delta_{i,j}, \quad i, j = 1 : K, \tag{65}$$

*or equivalently, the equations on $p_i$'s in* (64) *hold on* $\Omega \setminus (\bigcup_{i=1}^{K} Q_i)$ *with Neumann conditions along $\partial\Omega$, and Dirichlet conditions along* $\bigcup_{i=1}^{K} \partial Q_i : p_i|_{\partial Q_j} = \delta_{i,j}$.

Similarly, one has the following result for the piecewise constant SMS model (49), which carries much lower complexity compared with the full SMS model.

**Proposition 2** (Euler-Lagrange equations for piecewise constant SMS). *The Euler-Lagrange equations for $E[\mathbf{P}, \mathbf{m} \mid I]$ in* (49) *are given by*

$$\begin{aligned} m_i &= \langle I \rangle_{p_i} := \frac{\int_\Omega I p_i}{\int_\Omega p_i}, \\ -18\varepsilon\Delta p_i &+ 2\varepsilon^{-1} p_i(1-p_i)(1-2p_i) \\ &= \langle \mathbf{V} \rangle - \lambda(m_i - I)^2, \quad i = 1 : K, \end{aligned} \tag{66}$$

*with Neumann conditions for all the ownerships $p_i$'s along $\partial\Omega$.*

### 5.2. Computation of the Euler-Lagrange equations

Computationally, as well practiced in multivariate optimization problems, (64) and (66) can be solved via the algorithm of *alternating minimization* (AM) [30, 39]. The AM algorithm is closely connected to the celebrated expectation-maximization (EM) algorithm in statistical estimation problems with hidden variables [3, 43]. In the current context, the ownership distributions $p_i$'s could be treated as the hidden variables.

Like EM, the AM algorithm is progressive. Given the current ($t = n$) best estimation of the patterns $\mathbf{U}^n = (u_i^n \mid i = 1 : K)$, by solving

$$\mathbf{P}^n = \arg\min_{\mathbf{P}} E[\mathbf{P} \mid \mathbf{U}^n, I], \tag{67}$$

or equivalently,

$$\begin{aligned} -18\varepsilon\Delta p_i &+ 2\varepsilon^{-1} p_i(1-p_i)(1-2p_i) \\ &= \langle \mathbf{V}^n \rangle - \lambda(u_i^n - I)^2, \quad i = 1 : K, \end{aligned} \tag{68}$$

with Neumann boundary conditions, one obtains the current best estimation of the ownerships $\mathbf{P}^n = (p_i^n \mid i = 1 : K)$. Subsequently, based on $\mathbf{P}^n$, by solving

$$\mathbf{U}^{n+1} = \arg\min_{\mathbf{U}} E[\mathbf{U} \mid \mathbf{P}^n, I], \tag{69}$$

or equivalently,

$$-\alpha\Delta u_i + (\lambda p_i^n) u_i = (\lambda p_i^n) I, \quad i = 1 : K, \tag{70}$$

with Neumann boundary conditions, one completes a single round of pattern updating $\mathbf{U}^n \to \mathbf{U}^{n+1}$. The same procedure applies to the piecewise constant SMS equations in (66).

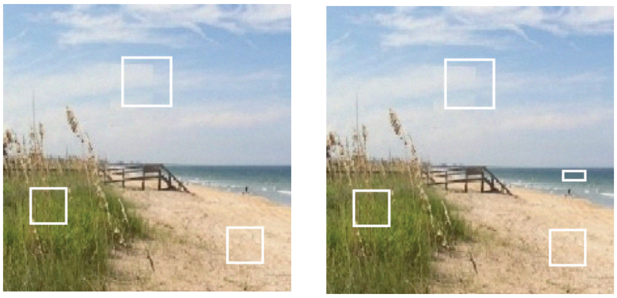

(a) (b)

Figure 2: Examples of (a) a 3-phase supervision and (b) a 4-phase supervision to break the symmetry in the model. Such weak supervision can also be automated based on multiscale patch statistics [1, 2].

Since the system (70) is linear and decoupled, the main computational complexity resides in the integration of (68), which is coupled and nonlinear due to the simplex constraint and the double-well potential in the energy. Define $e_i(x) = (u_i(x) - I(x))^2$ and $\mathbf{e} = (e_i \mid i = 1 : K)$. In order to solve

$$-18\varepsilon\Delta p_i + 2\varepsilon^{-1} p_i(1-p_i)(1-2p_i) = \langle \mathbf{V} \rangle - \lambda e_i \tag{71}$$

given $\mathbf{e}$ and $\mathbf{V} = \mathbf{V}(\mathbf{P}, \mathbf{U}) = \mathbf{V}(\mathbf{P}, \mathbf{e})$ (see (55)), first notice that

$$\begin{aligned} \langle \mathbf{V}(\mathbf{P}, \mathbf{e}) \rangle &= \frac{1}{K} \sum_{i=1}^{K} \left(-18\varepsilon\Delta p_i + \lambda e_i + 2\varepsilon^{-1} p_i(1-p_i)(1-2p_i)\right) \\ &= \frac{\lambda}{K} \sum_{i=1}^{K} e_i + \frac{2\varepsilon^{-1}}{K} \sum_{i=1}^{K} (2p_i^3 - 3p_i^2) + \frac{2\varepsilon^{-1}}{K}, \end{aligned} \tag{72}$$

since $\sum_{i=1}^{K} p_i = 1$ and $\Delta(\sum_{i=1}^{K} p_i) = 0$. We also split the double-potential force in (71) by

$$p_i(1-p_i)(1-2p_i) = p_i(1-p_i)^2 - p_i^2(1-p_i). \tag{73}$$

In combination, the nonlinear equation (71) can then be solved iteratively:

$$\cdots \longrightarrow \mathbf{P}^{\langle j \rangle} \longrightarrow \mathbf{P}^{\langle j+1 \rangle} \longrightarrow \cdots \tag{74}$$

by the following linearization procedure:

$$\begin{aligned} -18\varepsilon\Delta p_i^{\langle j+1 \rangle} + 2\varepsilon^{-1} p_i^{\langle j+1 \rangle} \left(1 - p_i^{\langle j \rangle}\right)^2 &= f_i^{\langle j \rangle}, \\ f_i^{\langle j \rangle} = -\lambda e_i + \langle \mathbf{V}(\mathbf{P}^{\langle j \rangle}, \mathbf{e}) \rangle + 2\varepsilon^{-1} \left(p_i^{\langle j \rangle}\right)^2 \left(1 - p_i^{\langle j \rangle}\right)&, \end{aligned} \tag{75}$$

with Neumann adiabatic boundary conditions for all the channels $i = 1 : K$. This system of linear Poisson equations can be conveniently integrated using any elliptic solvers. The detailed *numerical analysis* on the convergence behavior of the entire algorithm above, however, is still an open problem and well deserves some systematic investigation.

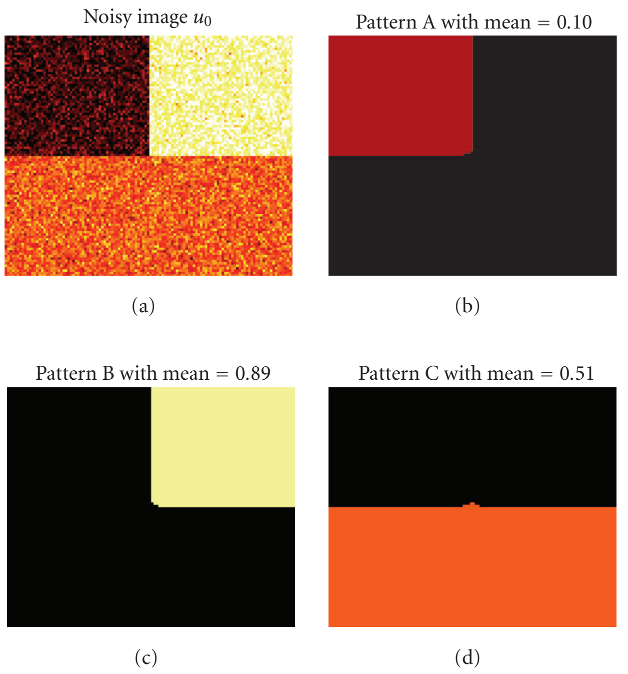

FIGURE 3: Synthetic image of a $T$-junction: hard segmentation from the SMS model via "hardening" formulae (5) and (6). The 120-degree regularization behavior at the junction point is also well known in the classical Mumford-Shah model [6].

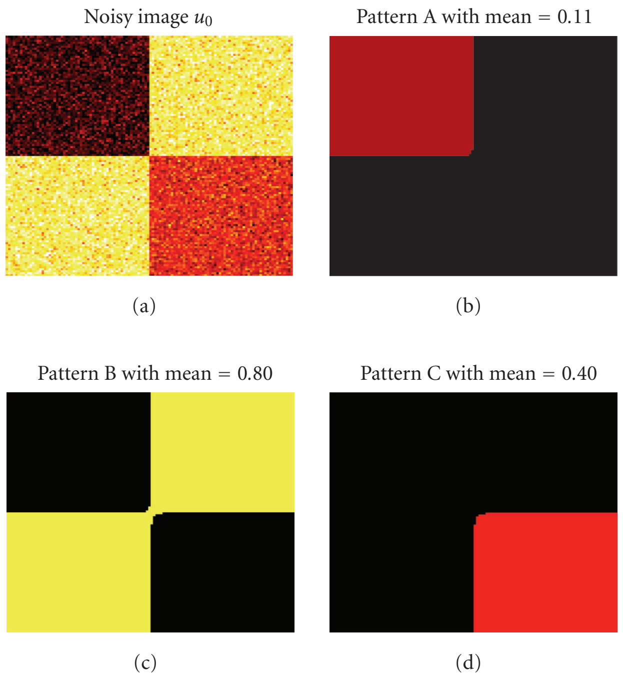

FIGURE 4: Synthetic image of a narrow bottleneck: hard segmentation from the SMS model via "hardening" formulae (5) and (6). The thickening regularization at the bottleneck junction can be explained similarly by the classical Mumford-Shah model for which minimum-surface or "soap-foam" behavior arises due to the surface tension energy. Also, see the recent work by Kohn and Slastikov [45] for the singularity analysis of a similar problem arising from micromagnetism.

### 6. COMPUTATIONAL EXAMPLES

In this section, we present the computational results of the proposed soft Mumford-Shah model. Notice that the extension of the above SMS models to color images is straightforward by having the gray values $u_i$'s replaced by RGB vectors. (We, however, must remind the reader that *perceptually* RGB may not be the most ideal representation of colors compared with other nonlinear approaches, e.g., brightness-chromaticity [42] and HSV [44].)

Figures 3 and 4 illustrate the performance of the SMS model on two synthetic images with multiple phases. Figure 3 shows a typical $T$-junction and Figure 4 shows a 3-phase image with a narrow bottleneck. Plotted in the figures are the hard segments obtained from the SMS model via the hardening formulae (5) and (6).

Plotted in Figures 5 and 6 are the hardened segments of two MRI brain images computed by the soft Mumford-Shah segmentation model via formulae (5) and (6). For this application, a user specifies three small patches (three rectangles in both examples) $Q_1$, $Q_2$, and $Q_3$, and the SMS model proceeds with the extra interpolation conditions in (46) for the ownerships. Notice in the second example that the detailed branching of the complex boundary is well resolved by the model.

In Figure 7, another example of a natural image is segmented via the SMS model and the "hardening" formulae (5) and (6). A user supervises with three patches $Q_1$, $Q_2$, and $Q_3$, and designates the two on the body to a pattern ownership $p_{\text{body}}$ and the third (from the ocean) to $p_{\text{ocean}}$. If the three are treated as distinct patterns, the SMS model still works, but one needs an extra step of high-level vision processing (e.g., based on Grenander's graph models [46]) to group the skin-tone and the purple-shirt patterns in order to capture the entire body faithfully.

Finally, plotted in Figures 8 and 9 are the ownerships from the SMS model based on the 3-phase and 4-phase supervisions separately in Figure 2. The stochastic nature of the outcomes (i.e., the softly transiting ownerships $p_i$'s instead of hard segmentation) is closer to the way a human subject may perceive such a natural scene. In particular, the SMS model seems to be consistent with the most recent theory that *hard* pattern segments may not be absolutely necessary for natural scene recognition [1, 2].

### 7. CONCLUSION

In this work, we have improved the celebrated Mumford-Shah segmentation to allow stochastic fuzziness of individual patterns. The proposed model outputs the ownership (or membership) probability distributions for all the patterns, from which the classical *hard* segmentation can be obtained based on stochastic decision rules such as the principle of maximum likelihood or the Bayesian classifier.

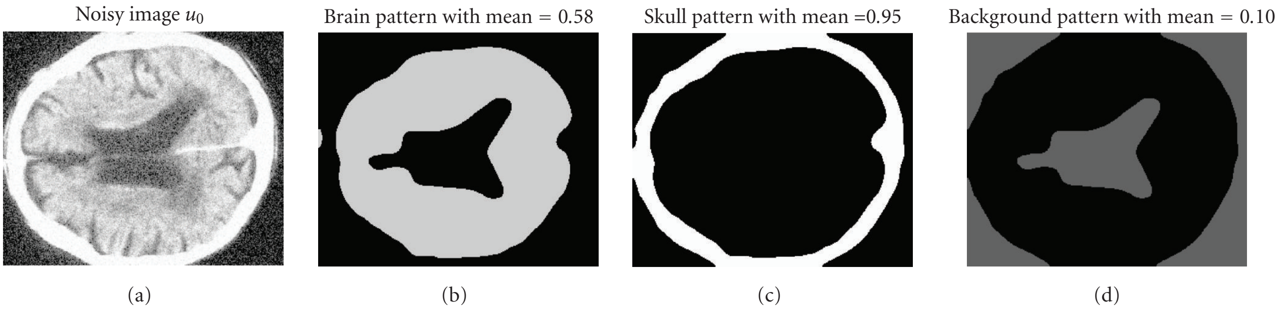

Figure 5: A real noisy brain image: hard segmentation from the SMS model via "hardening" formulae (5) and (6).

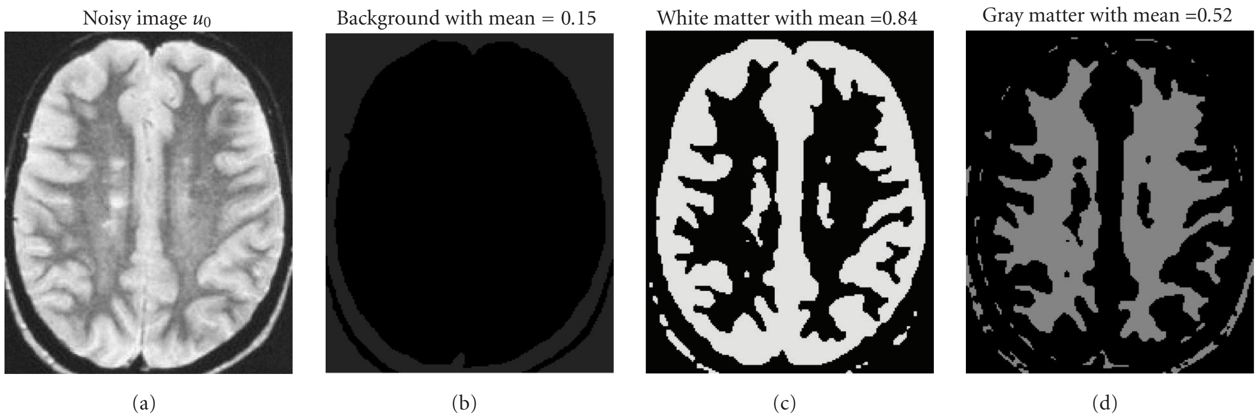

Figure 6: A brain image with low noise: hard segmentation from the SMS model via "hardening" formulae (5) and (6). Notice how the detailed branching of the gray matter has been successfully resolved by the model.

The key component of the new model is an ensemble of regularized double-well potentials inspired by the literature of material sciences and variational calculus. The model is nonconvex and the existence of optimal soft segmentation has been proven. A preliminary algorithm has been proposed and implemented, but without convergence analysis. Several generic numerical examples have demonstrated the flexibility and performance of the new model.

Our future work will mainly focus on (1) automating the weak supervision process based on statistical patch analysis, as inspired by the recent work of Li and Perona [1], and (2) developing a comprehensive framework for the effective computation of such a nonconvex and multivariate variational model (with Alan Yuille).

### APPENDIX

### PROOF OF THE EXISTENCE THEOREM 3

We will need the following lemma for the proof.

**Lemma 2.** *Let* $(f_n \mid n)$ *be a sequence of functions in* $L^2(\Omega)$, *and* $(p^n \mid n)$ *a sequence of nonnegative measurable functions on* $\Omega$ *and valued in* $[0,1]$. *Suppose that*

(i) $p^n \to p^*$, *a.e. on* $\Omega$, *and* $\int_\Omega p^* > 0$;

(ii) $\int_\Omega f_n^2 p^n \leq A$ *for some* $A > 0$ *and* $n = 1 : \infty$.

*Then there exists some function* $\rho \in L^2(\Omega)$, *such that*

(a) $\rho \geq 0$ *and* $\int_\Omega \rho = 1$;
(b) *for some fixed* $B > 0$, $|\int_\Omega f_n \rho| \leq B$ *for* $n = 1 : \infty$.

*Proof.* Denote the Lebesgue measure of a measurable set $W$ by $|W|$. Since $p^* \geq 0$ and $\int_\Omega p^* > 0$, there must exist some $c > 0$, such that

$$V = \{x \in \Omega \mid p^* > 2c\} \quad \text{has a finite but positive measure.} \tag{A.1}$$

On the other hand, by Egorov's theorem [47] on a.e. convergence, there must exist a subset $W \subset V$, such that

(a′) $|V - W| \leq |V|/2$, and hence $|W| > 0$;
(b′) $p^n \to p^*$ uniformly on $W$.

In particular, there exists some $N$, such that for any $n > N$, $p^n > c$ on $W$. Define

$$\rho(x) = \frac{1_W(x)}{|W|} \in L^2(\Omega). \tag{A.2}$$

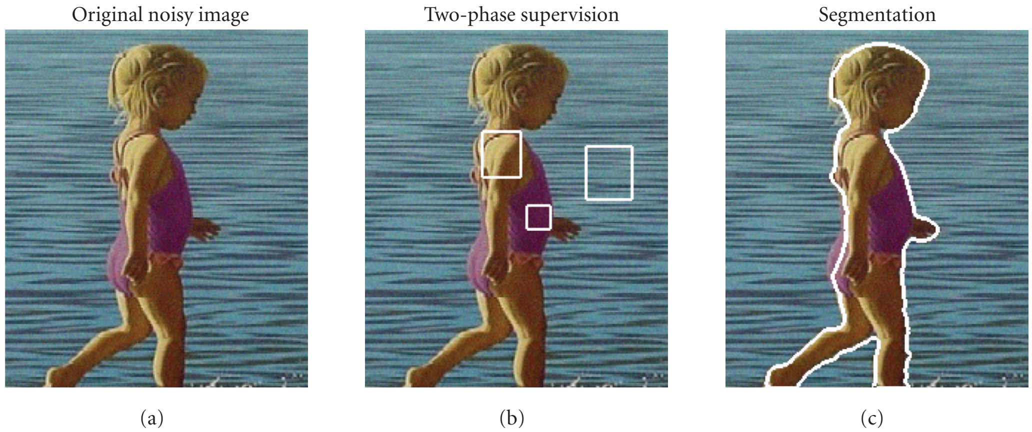

FIGURE 7: Hard segmentation from the SMS model via "hardening" formulae (5) and (6), based on a 2-phase supervision. Denote the two rectangles on the body by $Q_1$ and $Q_2$, and the third by $Q_3$. Supervision provides the ownership interpolation condition: $p_{\text{body}} = 1$ on $Q_1 \cup Q_2$ and 0 on $Q_3$, while $p_{\text{ocean}} = 1$ on $Q_3$ and 0 on $Q_1 \cup Q_2$. Patch selection can also be automated based on multiscale patch statistics (e.g., see Li and Perona [1]).

Then $\int_\Omega \rho = 1$, and for any $n > N$,

$$\int_\Omega f_n^2 \rho = \frac{1}{c|W|}\int_W f_n^2 c \le \frac{1}{c|W|}\int_\Omega f_n^2 p^n \le \frac{A}{c|W|}. \quad (A.3)$$

Thus by the Schwarz inequality (or $E[X]^2 \le E[X^2]$ in probability theory),

$$\left|\int_\Omega f_n \rho\right| \le \left(\int_\Omega f_n^2 \rho\right)^{1/2} \le \left(\frac{A}{c|W|}\right)^{1/2}, \quad n > N. \quad (A.4)$$

The lemma holds if one defines

$$B = \max\left(\left(\frac{A}{c|W|}\right)^{1/2}, \left|\int_\Omega f_1\rho\right|, \dots, \left|\int_\Omega f_N\rho\right|\right). \quad (A.5)$$

□

We are ready to prove Theorem 3.

*Proof.* Take the special pattern distribution:

$$u_i \equiv 0, \quad i = 1:K; \qquad p_1 \equiv 1, \qquad p_j \equiv 0, \quad j = 2:K. \quad (A.6)$$

Then

$$E[\mathbf{P}, \mathbf{U} \mid I] = \lambda \int_\Omega I^2 < \infty. \quad (A.7)$$

Thus the infimum of the energy must be finite. Let $(\mathbf{P}^n, \mathbf{U}^n \mid n) \subseteq \text{adm}_K$ (see (40)) be a minimizing sequence for the soft Mumford-Shah energy (37). That is, $E[\mathbf{P}^n, \mathbf{U}^n \mid I]$ converges to $\inf_{\mathbf{P},\mathbf{U}} E[\mathbf{P}, \mathbf{U} \mid I]$.

Due to the third term in the energy and the simplex constraint, for each channel $i$, $(p_i^n \mid n)$ must be bounded in $H^1(\Omega)$. By the $L^2$-weak compactness, there must exist some $\mathbf{P}^* \in L^2(\Omega, \mathbb{R}^K)$, and a subsequence of $(\mathbf{P}^n \mid n)$, which after relabelling will still be denoted by $(\mathbf{P}^n \mid n)$ for convenience, such that

$$\mathbf{P}^n \longrightarrow \mathbf{P}^* \quad \text{in } L^2(\Omega, \mathbb{R}^K),\ n \to \infty. \quad (A.8)$$

Then by the $L^2$ lower semicontinuity of Sobolev measures,

$$9\varepsilon \int_\Omega |\nabla p_i^*|^2 \le \liminf_{n\to\infty} 9\varepsilon \int_\Omega |\nabla p_i^n|^2, \quad i = 1:K. \quad (A.9)$$

Furthermore, with possibly another round of subsequence refinement, one can assume that

$$\mathbf{P}^n(x) \longrightarrow \mathbf{P}^*(x), \quad \text{a.e. } x \in \Omega,\ n \to \infty. \quad (A.10)$$

Since the probability simplex $\Delta_{k-1}$ is closed and $\mathbf{P}^n(x) \in \Delta_{K-1}$, one concludes that

$$\mathbf{P}^*(x) \in \Delta_{K-1}, \quad \text{a.e. } x \in \Omega. \quad (A.11)$$

And by Fatou's lemma [47, 48], one has

$$\int_\Omega \frac{(p_i^*(1-p_i^*))^2}{\varepsilon} \le \liminf_{n\to\infty} \int_\Omega \frac{(p_i^n(1-p_i^n))^2}{\varepsilon}, \quad i = 1:K. \quad (A.12)$$

(In fact, the equality holds by *Lebesgue's dominated convergence* [48].)

After the above subsequence selection on $\mathbf{P}^n$'s, one naturally has an associated subsequence of $(\mathbf{U}^n \mid n)$, which for convenience is still denoted by $(\mathbf{U}^n \mid n)$ after relabelling. For each *specific* channel $i$, we then consider two scenarios separately.

Suppose that $p_i^*(x) \equiv 0$, a.e. $x \in \Omega$. We then define for that channel

$$u_i^*(x) \equiv 0, \quad x \in \Omega. \quad (A.13)$$

Such a channel is called a "dumb" channel.

Otherwise, one must have $\int_\Omega p_i^* > 0$, and from the first term in (37),

$$\int_\Omega (u_i^n - I)^2 p_i^n \le \text{const}, \quad n = 1:\infty. \quad (A.14)$$

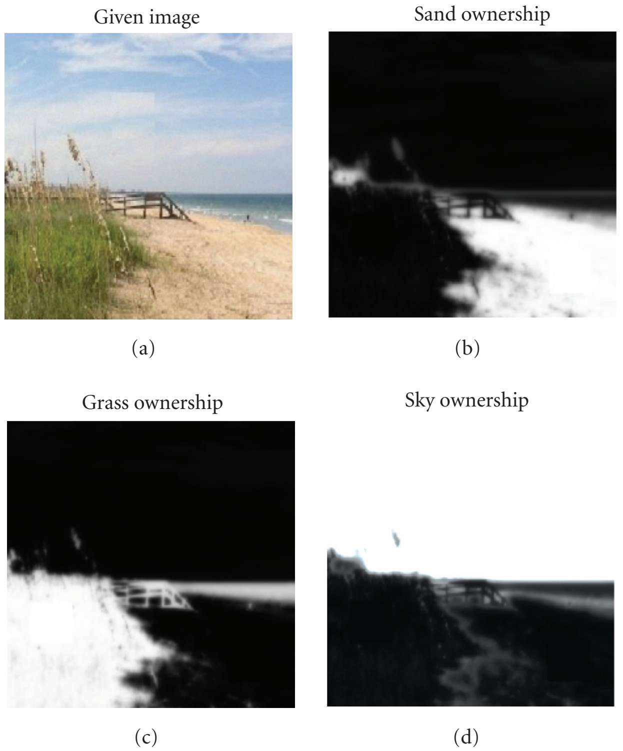

FIGURE 8: Soft Mumford-Shah segmentation with three phases corresponding to the supervision on Figure 2(a). Plotted here are the three ownership distributions $p_1(x), p_2(x)$, and $p_3(x)$. Due to "under"-supervision, namely the number $K$ of specified patterns is less than that of the visually meaningful ones, the grass pattern has "absorbed" the ocean pattern due to the greenish color they happen to share.

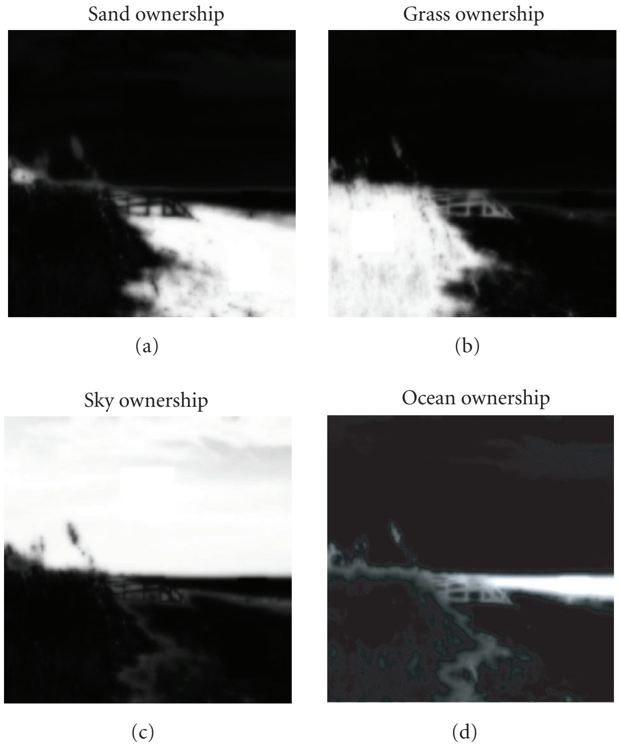

FIGURE 9: Soft Mumford-Shah segmentation with four phases corresponding to the supervision on Figure 2(b). Plotted here are the four ownership distributions $p_1(x), p_2(x), p_3(x)$, and $p_4(x)$. Unlike Figure 8, the narrow ocean pattern is now softly segmented due to the extra fourth patch $Q_4$.

Since $\int_\Omega I^2 p_i^n \leq \int_\Omega I^2$, by the triangle inequality,

$$\int_\Omega (u_i^n)^2 p_i^n \leq \text{const}, \quad n = 1 : \infty, \tag{A.15}$$

where the constant only depends on $I$ and the model parameters. Then by Lemma 2, there exists some $\rho_i(x) \geq 0$, with $\int_\Omega \rho_i = 1$, some constant $B_i > 0$ such that

$$\left| \int_\Omega u_i^n \rho_i \right| \leq B_i, \quad n = 1 : \infty. \tag{A.16}$$

On the other hand, by the second term in the energy (37),

$$\int_\Omega |\nabla u_i^n|^2 \leq C_i = C_i(I, \lambda, \alpha, \varepsilon), \quad n = 1 : \infty, \tag{A.17}$$

for some constant $C_i$ independent of $n$. Then by the generalized Poincaré inequality [48, 49] on $\Omega$,

$$\|w - \langle w, \rho_i \rangle\|_{L^2} \leq A_i \|\nabla w\|_{L^2}, \tag{A.18}$$

where $A_i = A_i(\rho_i, \Omega)$ is independent of $w \in H^1(\Omega)$, one concludes that

$$\|u_i^n\|_{L^2} \leq D_i = D_i(A_i, B_i, C_i), \quad n = 1 : \infty, \tag{A.19}$$

for some constant $D_i$. As a result, $(u_i^n \mid n)$ must be bounded in $H^1(\Omega)$. By the $L^2$-weak compactness of bounded $H^1$-sequences, there is a subsequence of $(u_i^n \mid n)$, for convenience still denoted by $(u_i^n \mid n)$ after relabelling, such that

$$u_i^n \longrightarrow u_i^* \in L^2(\Omega), \quad n \longrightarrow \infty, \tag{A.20}$$

converging in the sense of both $L^2$ and almost everywhere. Then by the lower semicontinuity,

$$\int_\Omega |\nabla u_i^*|^2 \leq \liminf_{n \to \infty} |\nabla u_i^n|^2. \tag{A.21}$$

Finally, since $u_i^n(x) \to u_i^*(x)$ and $p_i^n(x) \to p_i^*(x)$, a.e. $x \in \Omega$, Fatou's lemma gives

$$\int_\Omega (u_i^* - I)^2 p_i^* \leq \liminf_{n \to \infty} \int_\Omega (u_i^n - I)^2 p_i^n. \tag{A.22}$$

Combining both cases just analyzed above, we have established that

$$\lambda \sum_{i=1}^K \int_\Omega (u_i^* - I)^2 p_i^* + \alpha \sum_{i=1}^K \int_\Omega |\nabla u_i^*|^2 \leq \liminf_{n \to \infty} \lambda \sum_{i=1}^K \int_\Omega (u_i^n - I)^2 p_i^n + \alpha \sum_{i=1}^K \int_\Omega |\nabla u_i^n|^2. \tag{A.23}$$

Together with (A.9) and (A.12), this implies that

$$E[\mathbf{P}^*, \mathbf{U}^* \mid I] \le \liminf_{n\to\infty} E[\mathbf{P}^n, \mathbf{U}^n \mid I] = \inf_{(\mathbf{P},\mathbf{U})} E[\mathbf{P}, \mathbf{U} \mid I], \tag{A.24}$$

and hence $(\mathbf{P}^*, \mathbf{U}^*)$ must be a minimizer. (We must caution our reader that since index relabelling has been performed for a couple of times to simplify notations, this last sequence $(\mathbf{P}^n, \mathbf{U}^n)$ is not the one we have started with originally.) This completes the proof. □

### ACKNOWLEDGMENTS

The author is very grateful to Professor Alan Yuille for an enlightening discussion after the current work was first presented. For their generous teaching and continual inspiration, the author is always profoundly indebted to Professors Gil Strang, Tony Chan, Stan Osher, David Mumford, Jean-Michel Morel, and Stu Geman. The author must thank his wonderful former teacher, Professor Dan Kerstan at the Psychology Department of the University of Minnesota, for his first introduction on mixture image models and stochastic visual processing several years ago. The author also thanks the Institute of Mathematics and its Applications (IMA) and the Institute of Pure and Applied Mathematics (IPAM) for their persistent role in supporting this new emerging field. Finally, the author would like to dedicate this paper to his dear friends Yingnian Wu and Song-Chun Zhu for the unique friendship cultivated by the intellectually rich soil of vision and cognitive sciences. The generous help from our referees is also enormous. This work has been partially supported by the NSF (USA) under Grant no. DMS-0202565.

### REFERENCES

[1] F.-F. Li and P. Perona, "A Bayesian hierarchical model for learning natural scene categories," in *IEEE Computer Society Conference on Computer Vision and Pattern Recognition (CVPR '05)*, vol. 2, pp. 524–531, San Diego, Calif, USA, June 2005.

[2] F.-F. Li, R. VanRullen, C. Koch, and P. Perona, "Rapid natural scene categorization in the near absence of attention," *Proceedings of the National Academy of Sciences of the United States of America*, vol. 99, no. 14, pp. 9596–9601, 2002.

[3] A. D. Jepson and M. J. Black, "Mixture models for image representation," PRECARN ARK Project Technical Report ARK96-PUB-54, University of Toronto, Toronto, Ontario, Canda, March 1996.

[4] D. L. Pham, C. Xu, and J. L. Prince, "Current methods in medical image segmentation," *Annual Review of Biomedical Engineering*, vol. 2, pp. 315–337, 2000.

[5] J.-M. Morel and S. Solimini, *Variational Methods in Image Segmentation*, vol. 14 of *Progress in Nonlinear Differential Equations and Their Applications*, Birkhäuser, Boston, Mass, USA, 1995.

[6] D. Mumford and J. Shah, "Optimal approximations by piecewise smooth functions and associated variational problems," *Communications on Pure and Applied Mathematics*, vol. 42, pp. 577–685, 1989.

[7] T. F. Chan and J. Shen, *Image Processing and Analysis: Variational, PDE, Wavelet, and Stochastic Methods*, SIAM, Philadelphia, Pa, USA, 2005.

[8] P. Brémaud, *Markov Chains: Gibbs Fields, Monte Carlo Simulation, and Queues*, Springer, New York, NY, USA, 1998.

[9] S. Geman and D. Geman, "Stochastic relaxation, Gibbs distributions, and the Bayesian restoration of images," *IEEE Transactions on Pattern Analysis and Machine Intelligence*, vol. 6, pp. 721–741, 1984.

[10] Z. Tu and S. C. Zhu, "Image segmentation by data-driven Markov chain Monte Carlo," *IEEE Transactions on Pattern Analysis and Machine Intelligence*, vol. 24, no. 5, pp. 657–673, 2002.

[11] S. C. Zhu and A. Yuille, "Region competition: unifying snakes, region growing, and Bayes/MDL for multi-band image segmentation," *IEEE Transactions on Pattern Analysis and Machine Intelligence*, vol. 18, no. 9, pp. 884–900, 1996.

[12] D. C. Knill and W. Richards, *Perception as Bayesian Inference*, Cambridge University Press, Cambridge, UK, 1996.

[13] D. Mumford, "The Bayesian rationale for energy functionals," in *Geometry Driven Diffusion in Computer Vision*, pp. 141–153, Kluwer Academic, Dordrecht, The Netherlands, 1994.

[14] L. Modica and S. Mortola, "Un esempio di Gammaconvergenza," *Bollettino della Unione Matematica Italiana B*, vol. 5–14, no. 1, pp. 285–299, 1977.

[15] B. Sandberg, T. F. Chan, and L. Vese, "A level-set and Gabor-based active contour algorithm for segmenting textured images," CAM report 02–39, UCLA Department of Mathematics, Los Angeles, Calif, USA, 2002.

[16] S. C. Zhu, Y. N. Wu, and D. Mumford, "Minimax entropy principle and its applications to texture modeling," *Neural Computation*, vol. 9, pp. 1627–1660, 1997.

[17] R. A. Adams and J. J. F. Fournier, *Sobolev Spaces*, Academic Press, New York, NY, USA, 2nd edition, 2003.

[18] D. Chandler, *Introduction to Modern Statistical Mechanics*, Oxford University Press, New York, NY, USA, 1987.

[19] W. Gibbs, *Elementary Principles of Statistical Mechanics*, Yale University Press, New Haven, Conn, USA, 1902.

[20] G. Strang, *Introduction to Applied Mathematics*, Wellesley-Cambridge Press, Wellesley, Mass, USA, 1993.

[21] J. L. Ericksen, "Equilibrium theory of liquid crystals," in *Advances in Liquid Crystals*, pp. 233–299, Academic Press, New York, NY, USA, 1976.

[22] V. L. Ginzburg and L. D. Landau, "On the theory of superconductivity," *Soviet Physics JETP*, vol. 20, pp. 1064–1082, 1950.

[23] G. Dal Maso, *An Introduction to Γ-Convergence*, Birkhäuser, Boston, Mass, USA, 1992.

[24] L. Ambrosio and V. M. Tortorelli, "Approximation of functionals depending on jumps by elliptic functionals via Γ-convergence," *Communications on Pure and Applied Mathematics*, vol. 43, pp. 999–1036, 1990.

[25] L. Ambrosio and V. M. Tortorelli, "On the approximation of free discontinuity problems," *Bollettino della Unione Matematica Italiana B*, vol. 6-B, pp. 105–123, 1992.

[26] L. C. Evans and R. F. Gariepy, *Measure Theory and Fine Properties of Functions*, CRC Press, Boca Raton, Fla, USA, 1992.

[27] E. Giusti, *Minimal Surfaces and Functions of Bounded Variation*, Birkhäuser, Boston, Mass, USA, 1984.

[28] L. Rudin, S. Osher, and E. Fatemi, "Nonlinear total variation based noise removal algorithms," *Physica D*, vol. 60, no. 1–4, pp. 259–268, 1992.

[29] C. M. Bishop, *Neural Networks for Pattern Recognition*, Oxford University Press, New York, NY, USA, 1995.

[30] S. Esedoglu and J. Shen, "Digital inpainting based on the Mumford-Shah-Euler image model," *European Journal of Applied Mathematics*, vol. 13, pp. 353–370, 2002.

[31] R. March, "Visual reconstruction with discontinuities using variational methods," *Image and Vision Computing*, vol. 10, no. 1, pp. 30–38, 1992.

[32] R. March and M. Dozio, "A variational method for the recovery of smooth boundaries," *Image and Vision Computing*, vol. 15, no. 9, pp. 705–712, 1997.

[33] J. Shen, "Γ-convergence approximation to piecewise constant Mumford-Shah segmentation," in *Proceedings of the 7th international Conference on Advanced Concepts for Intelligent Vision Systems*, vol. 3708 of *Lecture Notes in Computer Science*, pp. 499–506, Antwerp, Belgium, September 2005.

[34] J. Shen, "On the foundations of vision modeling II. Mining of mirror symmetry of 2-D shapes," *Journal of Visual Communication and Image Representation*, vol. 16, no. 3, pp. 250–270, 2005.

[35] J. Shen and Y.-M. Jung, "Weberized Mumford-Shah model with Bose-Einstein photon noise," to appear in *Applied Mathematics and Optimization*.

[36] F. Cucker and S. Smale, "On the mathematical foundations of learning," *Bulletin of the American Mathematical Society*, vol. 39, no. 1, pp. 1–49, 2001.

[37] S. Smale and D.-X. Zhou, "Shannon sampling and function reconstruction from point values," *Bulletin of the American Mathematical Society*, vol. 41, pp. 279–305, 2004.

[38] G. Aubert and P. Kornprobst, *Mathematical Problems in Image Processing*, Springer, New York, NY, USA, 2001.

[39] J. Shen, "Bayesian video dejittering by BV image model," *SIAM Journal on Applied Mathematics*, vol. 64, no. 5, pp. 1691–1708, 2004.

[40] D. Mumford and B. Gidas, "Stochastic models for generic images," *Quarterly of Applied Mathematics*, vol. 59, pp. 85–111, 2001.

[41] T. F. Chan and L. A. Vese, "Active contours without edges," *IEEE Transactions on Image Processing*, vol. 10, no. 2, pp. 266–277, 2001.

[42] T. F. Chan and J. Shen, "Variational restoration of non-flat image features: models and algorithms," *SIAM Journal on Applied Mathematics*, vol. 61, no. 4, pp. 1338–1361, 2000.

[43] R. J. A. Little and D. B. Rubin, *Statistical Analysis with Missing Data*, John Wiley & Sons, New York, NY, USA, 2002.

[44] T. F. Chan, S.-H. Kang, and J. Shen, "Total variation denoising and enhancement of color images based on the CB and HSV color models," *Journal of Visual Communication and Image Representation*, vol. 12, no. 4, pp. 422–435, 2001.

[45] R. V. Kohn and V. V. Slastikov, "Geometrically constrained walls," to appear in Calculus of Variations and PDE.

[46] U. Grenander, *Lectures in Pattern Theory I, II and III*, Springer, Berlin, Germany, 1976–1981.

[47] G. B. Folland, *Real Analysis - Modern Techniques and Their Applications*, John Wiley & Sons, New York, NY, USA, 2nd edition, 1999.

[48] E. H. Lieb and M. Loss, *Analysis*, American Mathematical Society, Providence, RI, USA, 2nd edition, 2001.

[49] L. C. Evans, *Partial Differential Equations*, American Mathematical Society, Providence, RI, USA, 1998.

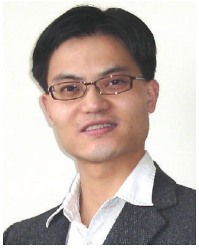

**Jianhong (Jackie) Shen** received the Ph.D. degree in applied mathematics from the Massachusetts Institute of Technology in 1998, and the B.S. degree from the University of Science and Technology of China (USTC) in 1994. He was a Computational and Applied Mathematics (CAM) Assistant Professor at University of California, Los Angeles (UCLA), from 1998 to 2000. He is currently an Assistant Professor of applied mathematics at the University of Minnesota, Minn, USA. His current research interests include image, signal, and information processing, vision modeling and computation, as well as multiscale and stochastic modeling in medical and biological sciences. His new book *Image Processing and Analysis—variational, PDE, wavelets, and stochastic methods,* coauthored with Professor Tony F. Chan (Dean of Physical Sciences, UCLA), has been published by the SIAM (Society for Industrial and Applied Mathematics) Publisher in September 2005. Most of his research activities can be found at http://www.math.umn.edu/~jhshen

